# Peritonitis after exposure to biocontrol-agent fumes containing *Talaromyces flavus:* a case report in peritoneal dialysis patient

**DOI:** 10.1186/s12882-022-02898-1

**Published:** 2022-08-09

**Authors:** Phanit Sookto, Talerngsak Kanjanabuch, Tamonwan Chamroensakchai, Nisa Thongbor, Somchai Eiam-Ong

**Affiliations:** 1 Department of Medicine, Sunpasitthiprasong Hospital, Ubon Ratchathani, Thailand; 2 Division of Nephrology, Department of Medicine, Faculty of Medicine, Chulalongkorn University, Bangkok, 10330 Thailand; 3 Center of Excellence in Kidney Metabolic Disorders, Faculty of Medicine, Chulalongkorn University, Bangkok, Thailand; 4 Dialysis Policy and Practices Program, School of Global Health, Faculty of Medicine, Chulalongkorn University, Bangkok, Thailand; 5 CAPD Excellent Center, King Chulalongkorn Memorial Hospital, Bangkok, Thailand

**Keywords:** *Talaromyces flavus*, Non-marneffei *Talaromyces*, Fungal peritonitis, Peritoneal dialysis, Biocontrol agents

## Abstract

**Background:**

The first case of *Taralomyces flavus* infection in human and peritoneal dialysis (PD) patient after exposure to biocontrol agent fumes is reported here.

**Case presentation:**

A 77-year-old Thai female farmer with kidney failure presented with peritonitis and PD catheter obstruction from fungal biofilms. The potential root cause of infection was associated with exposure to biocontrol-agent fumes containing pathogen during agricultural work in her garden. This source of infection has not been mentioned previously. Showering and changing clothes right after outdoor activity with a high density of fungal matters or dust should be added to the routine aseptic technique before performing PD bag exchange to prevent the system contamination. Although the patient received early treatment with liposomal amphotericin B, itraconazole, and catheter removal, according to the ISPD Guideline 2016 and the Global Guideline 2021, the outcome was unfavorable. Antifungal susceptibility testing later revealed that the pathogen was only susceptible to voriconazole. Thus, antifungal susceptibility should be tested if the patient fails or slowly responds to the primary antifungal regimen.

**Conclusions:**

*T. flavus* peritonitis is reported here after exposure to biocontrol-agent fumes containing the pathogen. This work also alerts and reiterates nephrology peers to be aware of this overlooked source of peritonitis, the exposure to dusty environments, specifically containing biocontrol-agent fumes.

## Background

Fungal peritonitis is a life-threatening condition in patients with peritoneal dialysis (PD) with an approximately 40% death rate [1]. Although *Talaromyces flavus* is the most common species amongst non-marneffei *Talaromyces* in nature, this species has never been reported causing human diseases. We report the first infected human after exposure to biocontrol-agent fumes containing the pathogen. The patient had an unfavorable outcome, albeit adequate treatment according to the International Society for PD (ISPD) 2016 [2] and Global Guideline for Rare Mould Infections 2021 of the European Confederation of Medical Mycology (ECMM) in cooperation with the International Society for Human and Animal Mycology (ISHAM) and the American Society for Microbiology (ASM) [3].

## Case presentation

**Fig. 1:**
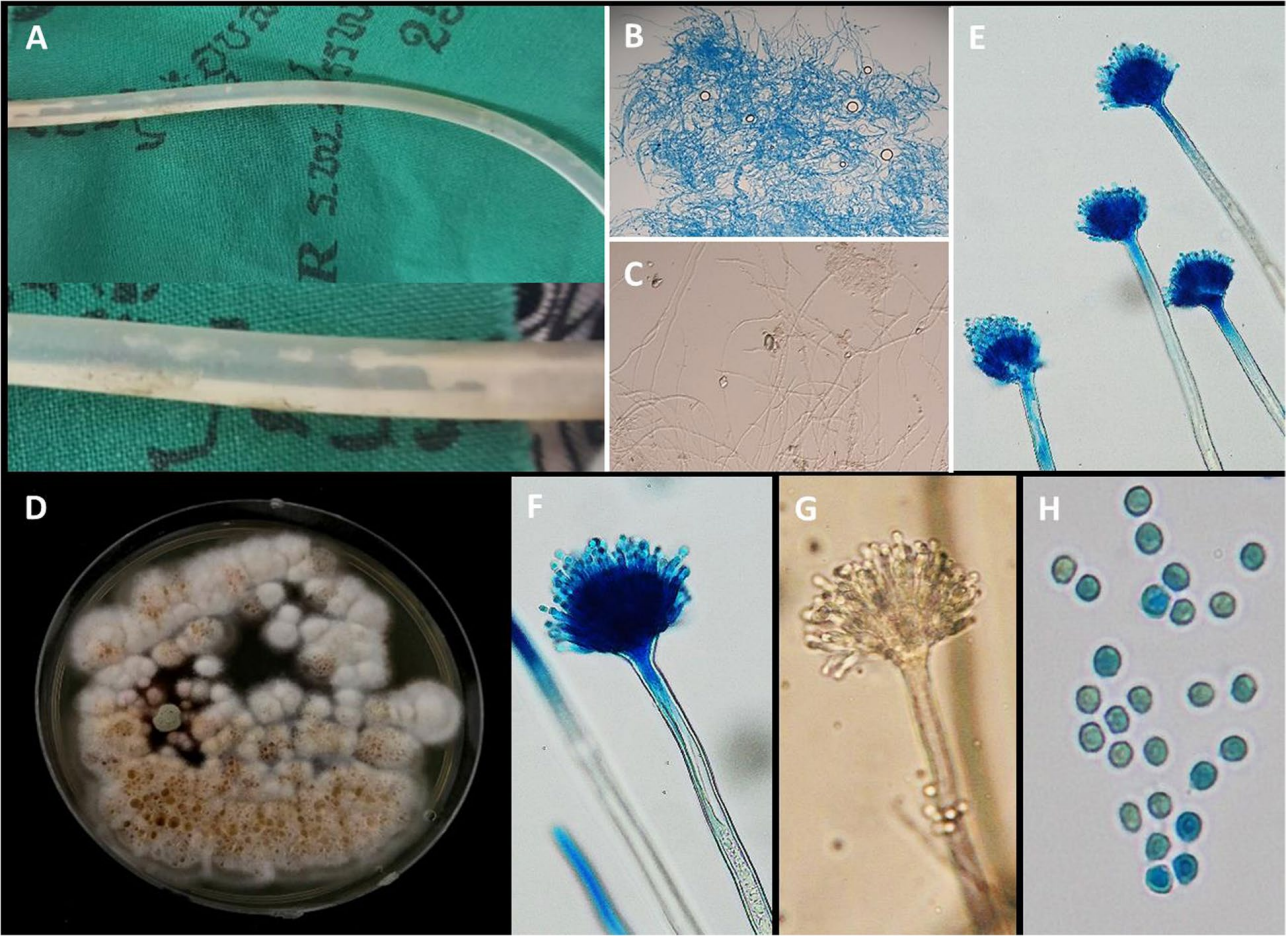
**A** Fungal colonization inside PD catheter lumen. Microscopic examination of the fungal colonization depicts fungal hyphae on Lactophenol blue (**B**) and KOH (**C**). Macroscopic finding on SDA, 25 °C after 14 days of incubation (**D**). Microscopic examination of the colonies demonstrated a classic feature of *the Penicillium* genus, including conidiophores and conidia (**E–H**) on wet smear (**G** × 400) and Lactophenol blue stains (**E** × 200, **F** × 400, **H** × 1000)

A 77-year-old Thai female with kidney failure on continuous ambulatory PD (1.5%D × 4 exchanges/day with ANDY.disc system since March 2013) presented at the PD clinic with acute abdominal pain and catheter malfunction on 17 June 2019 (day 0). Reviewing her PD logbook, negative to smaller positive ultrafiltration (-300 to + 100 mL) was disclosed in the past few days before this visit. No increased turbidity of the PD effluents had been observed before and during the onset of peritonitis. A plain abdomen radiogram revealed an excellent position of the PD catheter tip. Among testing the catheter function, numerous white colonization was observed inside the PD catheter lumen (Fig. 1A), the attending PD nurse stopped proceeding to the ‘push–pull’ syringe test. The catheter malfunction was confirmed with delayed both inflow and outflow rates. The dialysate cell counts disclosed numerous erythrocytes and leukocytes of 230/mm^3^ (35% neutrophils, 35% eosinophils, and 30% mononuclear cells). Clumping of filamentous hyaline and septate hyphae was detected by bedside wet smear (Fig. 1B and C); thus, fungal peritonitis was initially diagnosed. The PD catheter was removed, and temporary hemodialysis (HD) was initiated on day + 2 via a temporary hemodialysis (HD) catheter through the right internal jugular vein. The removed catheter was found to be partially occluded with fungal biofilm. Liposomal amphotericin B, 5 mg/kg daily, was started on day + 3, and continued for 14 days, followed by oral itraconazole, 400 mg daily (started on day + 17).

In microbiological exams of the PD effluent (PDE) and catheter, the colonies on Sabouraud dextrose agar (SDA) were initially white. However, after 14 days, they gradually developed a membranous aspect with floccose texture and started to darken, turning white to orange and brown (Fig. 1D). Microscopic examination of the colonies demonstrated classic features of *the Penicillium* genus, including conidiophores and conidia (Fig. 1E-H). The pathogen harvested from the colonies was confirmed the species of *T. flavus* by molecular phylogeny using ribosomal DNA (ITS and 28 s rDNA) sequences. The fungal susceptibility test was reported as an inconclusive result on D + 21 since there is no minimal inhibitory concentration (MIC) breakpoint value for *Talaromyces* from the European Committee on Antimicrobial Susceptibility Testing (EUCAST) [4]. Using *Aspergillus* spp. as a reference, the organism was considered resistance to all common use of antifungal medications (amphotericin B > 8 µg/mL, fluconazole 8 µg/mL, itraconazole > 8 µg/mL, caspofungin > 16 µg/mL) besides voriconazole < 0.5 µg/ml.

A root-cause analysis was performed. The patient was doing well with PD without a history of peritonitis in the past 2 years. She was a farmer and self-autonomy in performing PD exchanges and exit-site dressing. She could perform daily living activities without an assistant. The patient reported strict compliance with aseptic handwashing, wearing a mask, and PD exchange instructions. However, she reports fuming homemade biocontrol agents on her garden against soil-borne pathogens and rushing to perform PD bag exchange without changing her clothes on day -17. Nine days later (8 days before the peritonitis onset), she noticed tiny white spots inside her PD catheter, but she did not report to the treatment team. Cultures were performed from white/brown spots inside her PD bag exchange room, storage room, and bathroom, as well as the patient's hands, nail dirt, and the biocontrol agent. Only the biocontrol agent was positive with *T. flavus,* confirming a potential source of the infection. Of note, the patient used non-antiseptic soap solution for handwashing. Although the traditional soap, a salt of dodecyl sulfate, can eradicate bacteria from the washed skin, it has limited activity against fungi and mycobacterium. Antiseptic agent, retraining, and hand hygiene had been repeatedly emphasized.

**Fig. 2:**
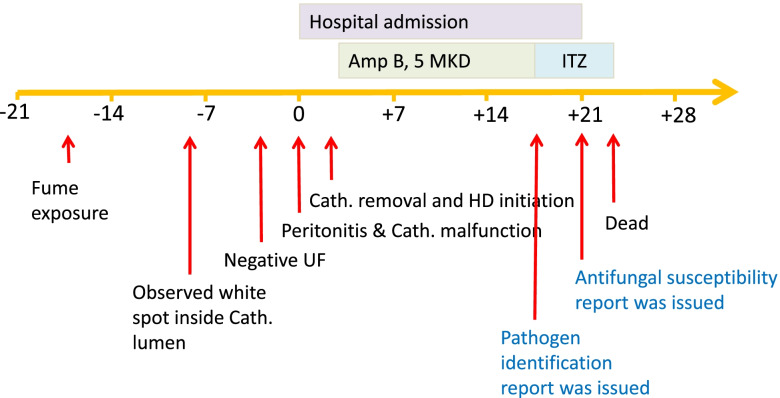
The patient’s clinical course. Abbreviations: Amp, amphotericin B; Cath, PD catheter; HD, hemodialysis; ITZ, itraconazole; MKD, milligram/kilogram body weight/day; and UF, ultrafiltration

She was discharged from the hospital on day + 21 with partial response to the antifungal medications, as shown by the modified Edmonton Symptom Assessment System score decreasing from 20 at the onset of infection to 7 on the discharge date (day + 21). On the discharge date, she was afebrile and had mild hypertension (blood pressures 140–150/80–90 mmHg), moderate anorexia, and constipation (Bristol stool scale of 1–2 and passing stool every 2–3 days) without nausea/vomiting. The patient was rescheduled for an HD visit in the next 3 days; however, the patient passed away at home 1 day before the appointment (day + 23) from unknown etiology. Her death was classified as peritonitis-associated death according to the 2022 ISPD Peritonitis Guidelines (defined as death occurring within 30 days of peritonitis onset or death during hospitalization due to peritonitis) [5]. The patient’s clinical course is demonstrated in Fig. 2.

## Discussion and conclusions

The first case of *T. flavus* infection in humans and PD is reported here with an unfavorable outcome, albeit an adequate treatment following the ISPD 2016 [2] and the Global Guideline 2021 [3]. The patient died of PD-related peritonitis during a course of antifungal medication. The patient was presented with peritonitis and catheter clogging from fungal biofilms. Catheter malfunction or obstruction has been occasionally reported in patients with peritonitis, usually from fibrin clots and rarely from fungal pathogen [6, 7]. Table 1 presents differential diagnoses of causative organisms of peritonitis in patients with specific features or exposures. The root-cause analysis revealed that exposure to homemade biocontrol-agent fumes containing the pathogen immediately before performing routine bag exchanges was the potential source of this infection.

**Table 1:** Differential diagnoses of causative organisms of peritonitis in patients with specific features or exposures

**With catheter malfunction**	- Fibrin clot (various bacteria with serious peritonitis) - Catheter entrapment (EPS, long-standing peritonitis, etc.) - Fungal colonization
**Visible catheter colonization**	- Fungal biofilm (filamentous molds [larger variably-colored, with hair-like diffuse edges, and central foci], yeast [usually white to off-white smaller translucent with defined edges], etc.) - Bacterial biofilms (staphylococci, non-fermenting Gram-negative bacterium, *Berholderia, Rhodococcus*)
**Exposure to biocontrol agents**	- Bacteria (*Agrobacterium, Bacillus, Pseudomonas, Streptomyces*, etc.) - Fungi (*Candida, Coniothyrium, Trichoderma*, *Talaromyces flavus, Cylindrocarpon destructans, Fusarium oxysporum, Rhizoctonia solani, Sclerotinia nivalis, Botrytis cinerea, Phytophthora capsici,* etc*.)*
**Pet-related**	- *Pasteurella multocida* - *Bordetella bronchiseptica* - *Capnocytophagia*
**Marine-related**	- *Aeromonas* spp. - *Edwardsiella tarda* - *Erysipelothrix* spp. - *V. vulnificus* - *Mycobacterium marinum* - *Shewanella* spp.
**Poor Oral hygiene**	- Viridans streptococci - *Fusobacterium* - *Actinomyces* - *Hemophilus*

*T. flavus* has been well documented and widely distributed in soil in the Northeastern region of Thailand [8]-where her house was located and has been frequently found in biocontrol agents against many soil-borne pathogens in tomato, cotton, cucumber, eggplant, and potato farms [9] as demonstrated in this case. Due to the unique exposure of the patient before this infectious episode, fumes contamination was a potential source of the infection. Showering and changing clothes right after outdoor activity with a high density of fungal matter, dust, or hay should be added to the routine aseptic technique before performing the bag exchange to prevent the PD system contamination. The ISPD Guidelines [10] do not mention this point during routine PD bag exchanges and only state that hand hygiene is critical in reducing infectious risk related to the performance of a PD exchange [10, 11]. Pathogens spill out from the patient's contaminated clothes or hairs is overlooked by the clinicians as a potential source of the infection. Indeed, it is generally accepted in the PD society that showering and changing clothes is unnecessary. This may not generalize to the case, returning from outdoor exposure to dusty environments or high airborne fungal load settings, as presented in our case. Thus, peritonitis may be preventable if the healthcare personnel is aware of and educates the patient about this potential route of infection.

Due to the lack of a specific treatment for *T. flavus* peritonitis, MIC breakpoint of this organism, and its rarity, liposomal amphotericin B at a dose of 5 mg/kg daily administration for 2 weeks followed with oral itraconazole after immediate catheter removal according to the ISPD 2016 Guideline [2] and the Global Guideline 2021 was unsuccessful in treating this infection, resulting in patient demise. Not only does itraconazole exhibits poor peritoneal penetration [12], but it also has limited affinity against this organism, as demonstrated in this case. Thus it should be avoided if treating *T. flavus* peritonitis. The organism is considered multi-antifungal resistant in nature; therefore, antifungal susceptibility testing should be encouraged to deal with an environmental pathogen. If the patients are not responding well to the first-line antifungal treatment, the choice of salvage antifungal agents should rely on the result of the antifungal susceptibility. Additionally, an antiseptic agent should be encouraged to use when washing hands in performing PD bag exchanges since traditional soap solution has limited activity in eradicating fungi and mycobacterium.

In conclusion, *T. flavus* should call for medical importance. Not only is it common in nature, but it is also multi-antifungal resistant, causing a fatal outcome despite immediate treatment and diagnosis. Antifungal susceptibility should be tested in patients with primary treatment failure or slow response to an empirical antifungal regimen. This work also alerts and reiterates nephrologists and allied health professional to be aware of an overlooked source of peritonitis, exposure to a dusty environment, specifically containing biocontrol-agent fumes.

## Data Availability

The data that support the findings of this study are available from the corresponding author upon reasonable request.

## Abbreviations

ASM: American Society for Microbiology
ECMM: European Confederation of Medical Mycology
EUCAST: European Committee on Antimicrobial Susceptibility Testing
HD: Hemodialysis
ISHAM: International Society for Human and Animal Mycology
ISPD: International Society for Peritoneal Dialysis
MIC: Minimal inhibitory concentration
PD: Peritoneal dialysis
PDE: PD effluent
SDA: Sabouraud dextrose agar
